# Controllable Assembly of Vanadium-Containing Polyoxoniobate-Based Materials and Their Electrocatalytic Activity for Selective Benzyl Alcohol Oxidation

**DOI:** 10.3390/molecules27092862

**Published:** 2022-04-30

**Authors:** Xiaoxia Li, Ni Zhen, Chengpeng Liu, Di Zhang, Jing Dong, Yingnan Chi, Changwen Hu

**Affiliations:** Key Laboratory of Cluster Science of Ministry of Education, Beijing Key Laboratory of Photoelectronic/Electrophotonic Conversion Materials, School of Chemistry and Chemical Engineering, Beijing Institute of Technology, Beijing 102488, China; 3120191243@bit.edu.cn (X.L.); zhenni1220@163.com (N.Z.); laucouple@163.com (C.L.); dizhang_pub@163.com (D.Z.); 20200808@btbu.edu.cn (J.D.)

**Keywords:** vanadium-containing polyoxoniobates, electrocatalysis, benzyl alcohol oxidation, nickel complexes

## Abstract

During the controllable synthesis of two vanadium-containing Keggin-type polyoxoniobates (PONbs), [Ni(en)_2_]_5_[PNb_12_O_40_(VO)_5_](OH)_5_·18H_2_O (**1**) and [Ni(en)_3_]_5_[PNb_12_O_40_(VO)_2_]∙17H_2_O (**2**, en = ethylenediamine) are realized by changing the vanadium source and hydrothermal temperature. Compounds **1** and **2** have been thoroughly characterized by single-crystal X-ray diffraction analysis, FT-IR spectra, X-ray photoelectron spectrum (XPS), powder X-ray diffraction (PXRD), etc. Compound **1** contains a penta-capped Keggin-type polyoxoniobate {PNb_12_O_40_(VO)_5_}, which is connected by adjacent [Ni(en)_2_]^2+^ units into a three-dimensional (3D) organic-inorganic framework, representing the first nickel complexes connected vanadoniobate-based 3D material. Compound **2** is a discrete di-capped Keggin-type polyoxoniobate {PNb_12_O_40_(VO)_2_} with [Ni(en)_3_]^2+^ units as counter cations. Compounds **1** and **2** have poor solubility in common solvents and can keep stable in the pH range of 4 to 14. Notably, both **1** and **2** as electrode materials are active for the selective oxidation of benzyl alcohol to benzaldehyde. Under ambient conditions without adding an alkaline additive, compound **1** as a noble metal free electrocatalyst can achieve 92% conversion of benzyl alcohol, giving a Faraday efficiency of 93%; comparatively, **2** converted 79% of the substrate with a Faraday efficiency of 84%. The control experiments indicate that both the alkaline polyoxoniobate cluster and the capped vanadium atoms play an important role during the electrocatalytic oxidation process.

## 1. Introduction

Polyoxoniobates (PONbs), as a unique branch of polyoxometalates (POMs), have drawn widespread attention in the past few decades due to their diverse structures and multiple applications in catalysis, nuclear-waste treatment, and virology [1,2]. Nevertheless, compared with other POM members, such as polyoxotungstates, polyoxomolybdates, and polyoxovanadates, the development of PONbs is relatively slow due to the lack of soluble Nb precursors, and their low reactivity and narrow working pH range [3]. Recently, great progress has been made in isopolyoxoniobates and some large clusters, such as {Nb_27_O_76_}, {Nb_32_O_96_}, {Nb_52_O_150_}, {Nb_81_O_225_}, {Nb_114_O_316_}, and the highest nuclearity {Nb_288_O_768_(OH)_48_(CO_3_)_12_} have been reported [4,5,6,7]. In 2002, the first Keggin-type PONb {(Ti_2_O_2_)SiNb_12_O_40_} was successfully synthesized by Nyman et al., marking the beginning of heteropolyoxoniobate chemistry [8]. After that, a series of heteropolyoxoniobates were reported and the Keggin-type {XNb_12_O_40_}^n−^ (X = Si, Ge, P) are the most extensively studied [9,10].

In the periodic table, Nb and V are neighbors with similar ionic radius and electronegativity, and their hydrolysis and condensation can be performed under alkaline conditions. Inspired by these similarities, in 2011, we synthesized the first Keggin-type vanadium-containing PONb {VNb_12_O_40_(VO)_2_} stabilized by Cu complex units [11]. Since then, a series of vanadium-containing Keggin-type PONbs and their derivatives have been reported, including {XNb_12_O_40_(VO)_2_} (X = Si, Ge, P, V), {XNb_12_O_40_(VO)_4_} (X = As, V), {PNb_10_V_2_O_40_(VO)_4_}, {XNb_12_O_40_(VO)_6_} (X = P, V), {VNb_14_O_42_L_2_} (L = CO_3_^2−^, NO_3_^−^), {XNb_8_V_4_O_40_(VO)_4_} (X = P, V, As), {AsNb_9_V_3_O_40_(VO)_4_}, {TeNb_9_V_2_O_37_}, and {TeNb_9_V_3_O_39_}, where the introduced V acts as central, capping, or/and substituted atoms [12,13,14,15,16,17,18,19,20,21,22,23]. Notably, most of the clusters were modified by metal-complex units. The use of the metal-complex unit not only contributes to the isolation of novel Keggin-type PONbs, but can also link the discrete PONb clusters into extended structures. In general, most transition metals tend to hydrolyze rapidly into precipitation under alkaline conditions, and thereby the coexistence of transition metal ions with basic PONbs is a challenge [3]. As the Cu ion can tolerate the alkaline synthesis conditions combining with its Jahn–Teller effect, Cu-complexes are the dominated metal organic units in the synthesis of PONb-based hybrids [24,25,26,27,28]. In contrast, Ni-complexes were seldom used and the extended structure based on V-containing PONb hybrids and a Ni-complex is rare. 

Compared with other POM members, the catalytic properties of PONbs are not extensively explored. Due to their Brønsted basicity, PONbs have been used to promote the hydrolysis of chemical warfare agents [29,30]. The introduction of V endows basic PONb clusters with interesting redox properties. For example, a double-anion cluster {PNb_12_O_40_(VO)_2_(V_4_O_12_)_2_} was successfully prepared in our group, which can effectively promote the basic hydrolysis of the nerve agent simulant and the oxidative decontamination of the sulfur mustard simulant [31]. Then, we found that the organic-inorganic hybrids based on {PNb_12_O_40_(VO)_2_} were active for the selective oxidation of benzyl-alkanes to ketone [14]. Our investigation indicates that the V atoms of {V_5_Nb_23_O_80_} and {V_6_Nb_23_O_81_} play a key role in the selective oxidation of the sulfur mustard simulant [32]. Owing to their fast and reversible electron transfer behavior, POMs are also a kind of promising electrocatalyst [33,34,35]. Recently, the covalent triazine framework immobilized {PMo_10_V_2_O_40_} shows excellent activities in the electrocatalytic oxidation of benzyl alcohols and ethylbenzene [36,37]. However, the electrocatalytic activity of vanadium-containing PONbs is nearly unexplored.

**Scheme 1:**
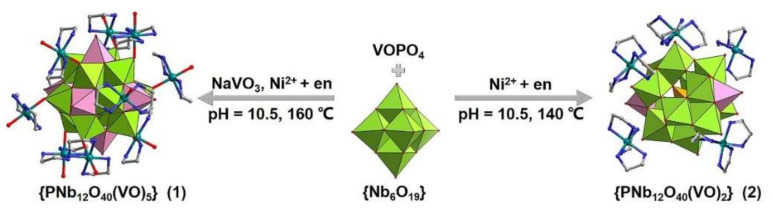
Controllable synthesis of **1** and **2**.

Herein, we report the controllable synthesis and structural characterization of two vanadium-containing Keggin-type PONbs: [Ni(en)_2_]_5_[PNb_12_O_40_(VO)_5_](OH)_5_·18H_2_O (**1**) and [Ni(en)_3_]_5_[PNb_12_O_40_(VO)_2_]∙17H_2_O (**2**, en = ethylenediamine) (Scheme 1). Compound **1** contains a Keggin-type PONb capped by five vanadyl groups, which was further connected by [Ni(en)_2_]^2+^ units into a three-dimensional (3D) organic-inorganic framework. Compound **2** is a di-capped discrete vanadoniobate cluster with a [Ni(en)_3_]^2+^ unit as count cations. Interestingly, **1** and **2** as electrode materials can catalyze the selective oxidation of benzyl alcohol to benzaldehyde under alkaline additive free conditions and compound **1** is more active than **2**. The control experiments show that both the capped V atom and the basic PONb cluster contribute to the enhancement of electrocatalytic activity. 

## 2. Experimental Section

### 2.1. Materials and Methods

The hexaniobate precursor K_7_HNb_6_O_19_·13H_2_O, vanadyl phosphate VOPO_4_·2H_2_O, TMA_10_H_5_[PNb_12_O_40_]·30.5H_2_O, K_6_[V_10_O_28_]·10H_2_O, and TMA_9_[PNb_12_V_2_O_42_]·19H_2_O were prepared according to previous literature methods and identified by IR spectroscopy [10,38,39,40,41]. Other starting chemicals and solvents were purchased from commercial source and used without further purification. IR spectra in KBr pellets were collected in the range of 400–4000 cm^−1^ using a Bruker FT-IR spectrometer (Leipzig, Germany). Thermogravimetric analyses (TGA) of the compounds were performed using a LABSYS EVO device (Setaram Inc., Lyon, France) from room temperature to 800 °C under N_2_ atmosphere. Elemental analyses (Nb, V, Ni, P) were measured on a Thermo ICP atomic emission spectrometer (Waltham, MA, USA); C, H, N were performed on an ElementarVario EL cube Elmer CHN elemental analyzer (Langenselbold, Germany). X-ray photoelectron spectrum (XPS) analysis were measured on a Thermo ESCALAB 250 spectrometer using Al Kα radiation as the X-ray source (1486.7 eV). Powder X-ray diffraction (PXRD) data were obtained on SHIMADZU XRD-6000 X-ray diffractometer (Kyoto, Japan) with Cu Kα radiation (λ = 1.54 Å; 2θ = 5–50°). Gas chromatograph analyses were detected on a Shimadzu GC-2014C instrument with an FID detector equipped with an HP 5 ms capillary column. The hydrogen was detected by a Techcomp GC-9700 gas chromatograph (Shanghai, China) with a 5 Å molecular sieve column (2 m × 2 mm) and a thermal conductivity detector (TCD). Temperature-programmed chemisorption of carbon dioxide (CO_2_-TPD) was performed on a PCA-1200 temperature-programmed chemisorption instrument. The UV-vis spectra were measured on a UV-2600 (Builder, Beijing, China). Electrochemical surface area experiments were measured on a CHI660E electrochemical workstation (CH Instruments, Shanghai, China). Other electrochemical experiments were performed on an Ivium-OctoStat30 multi-channel electrochemical workstation (Eindhoven, The Netherlands).

### 2.2. Synthesis of [Ni(en)_2_]_5_[PNb_12_O_40_(VO)_5_](OH)_5_·18H_2_O *(**1**)*

Ni(CH_3_COO)_2_·4H_2_O (0.2240 g, 0.9 mmol) was dissolved in distilled water (0.83 mL), and to this en (0.13 mL) was added, obtaining a purple solution. Then the resulting solution was added dropwise to K_7_HNb_6_O_19_·13H_2_O (0.1507 g, 0.11 mmol) aqueous solution (8 mL) containing VOPO_4_·2H_2_O (0.0534 g, 0.27 mmol) and NaVO_3_ (0.0329 g, 0.27 mmol) under stirring. The pH value of the mixture was adjusted to 10.50 using 2 M NaOH. The mixture was transferred to a Teflon-lined autoclave (23 mL) and kept at 160 °C for 72 h, and then slowly cooled to room temperature after 24 h, and brown block crystals of **1** were isolated in about 17.5% yield (based on Nb). Anal. Calcd (%) for **1**: C, 7.02; H, 3.56; N, 8.18; Ni, 8.57; P, 0.90; V, 7.44; Nb, 32.56. Found: C, 7.57; H, 3.47; N, 7.83; Ni, 8.06; P, 0.48; V, 7.05; Nb, 32.34. FT-IR (cm^−1^): 3451 (s), 2947 (w), 2883 (w), 1589 (s), 1460 (m), 1395 (w), 1330 (w), 1282 (s), 1233 (w), 1112 (w), 1032 (s), 942 (s), 870 (s), 805 (w), 700 (s), 627 (w), 498 (w), 473 (w).

### 2.3. Synthesis of [Ni(en)_3_]_5_[PNb_12_O_40_(VO)_2_]∙17H_2_O *(**2**)*

Compound **2** was synthesized by a similar procedure to that of **1**, but without adding NaVO_3_. The pH value of the mixture was adjusted to 10.50 using 2 M NaOH and transferred to a Teflon-lined autoclave (23 mL), kept at 140 °C for 72 h, and then slowly cooled to room temperature. Brown block crystals of **2** were isolated in about 21.8% yield (based on Nb). Anal. Calcd (%) for **2**: C, 10.53; H, 4.54; N, 12.28; Ni, 8.58; P, 0.91; V, 2.98; Nb, 32.59. Found: C, 10.46; H, 4.42; N, 11.84; Ni, 8.31; P, 0.64; V, 2.77; Nb, 32.86. FT-IR (cm^−1^): 3425 (s), 2945 (w), 2875 (w), 1593 (s), 1469 (m), 1363 (w), 1318 (w), 1275 (s), 1233 (w), 1106 (w), 1027 (s), 947 (s), 877 (s), 815 (w), 709 (s), 638 (w), 505 (w), 469 (w).

### 2.4. Preparation of Working Electrode

Grinded crystal samples of compound **1** or **2** (10 mg) and acetylene black (3 mg) were dispersed uniformly in isopropanol (0.5 mL) containing 5 wt% Nafion under ultrasonic conditions. A total of 50 μL of the suspension was drop-cast onto a piece of carbon cloth (1 cm^2^) and then dried slowly at room temperature.

### 2.5. Cyclic Voltammetry Experiments

Cyclic voltammetry experiments were performed in acetonitrile (10 mL) containing supporting electrolyte LiClO_4_ (1.0 mmol) and benzyl alcohol (0.5 mmol) under ambient conditions, and the scan rate was kept at 40 mV s^−1^. The cyclic voltammetry tests of **1** and **2** were performed using a three-electrode setup: carbon cloth modified by **1** or **2** as the working electrode, platinum plate electrode as the counter electrode, and Ag/Ag^+^ as the reference electrode.

### 2.6. Electrochemical Surface Area Experiments

Electrochemical surface areas of compounds **1** and **2** were estimated by the capacitance of the double layer *C*_dl_, which were determined by cyclic voltammetry tests [42]. For the cyclic voltammetry tests of **1** and **2**, glassy carbon electrode (3 mm diameter) was dripped with 5 μL isopropanol suspension of **1** or **2**, and served as the working electrode. The potential window was 0.01–0.13 V vs. Ag/Ag^+^, where no Faradaic processes occur. The scan rates were 10 mV s^−1^, 20 mV s^−1^, 30 mV s^−1^, 40 mV s^−1^, 50 mV s^−1^, 60 mV s^−1^, 70 mV s^−1^, 80 mV s^−1^, 90 mV s^−1^, and 100 mV s^−1^. The *C*_dl_ was calculated by plotting the relationship between Δ*j* and scanning rate at 0.07 V (Δ*j* = *j*_a_ − *j*_c_, *j*_a_, and *j*_c_ represent the current densities of the anode and cathode, respectively), and the slope of the image is twice that of *C*_dl_.

### 2.7. Controlled Potential Electrolysis Experiments

Bulk electrolysis experiments were performed in an undivided cell using a three-electrode setup with carbon cloth modified by catalysts as the working electrode, platinum plate electrode as the counter electrode, and Ag/Ag^+^ electrode as the reference electrode. A mixture of acetonitrile (10 mL) containing supporting electrolyte LiClO_4_ (1.0 mmol) and benzyl alcohol (0.5 mmol) was added to the undivided cell with applied potential of 1.6 V vs. Ag/Ag^+^. After the electrolysis experiment, biphenyl was added to the reaction solution as internal standard, and then the product was quantitatively detected by GC. For the recycle test, carbon cloth modified by compound **1** was washed three times with acetonitrile and ethyl alcohol, and dried for the next cycle.

## 3. Results and Discussion

### 3.1. Synthesis and Structure

**Figure 1:**
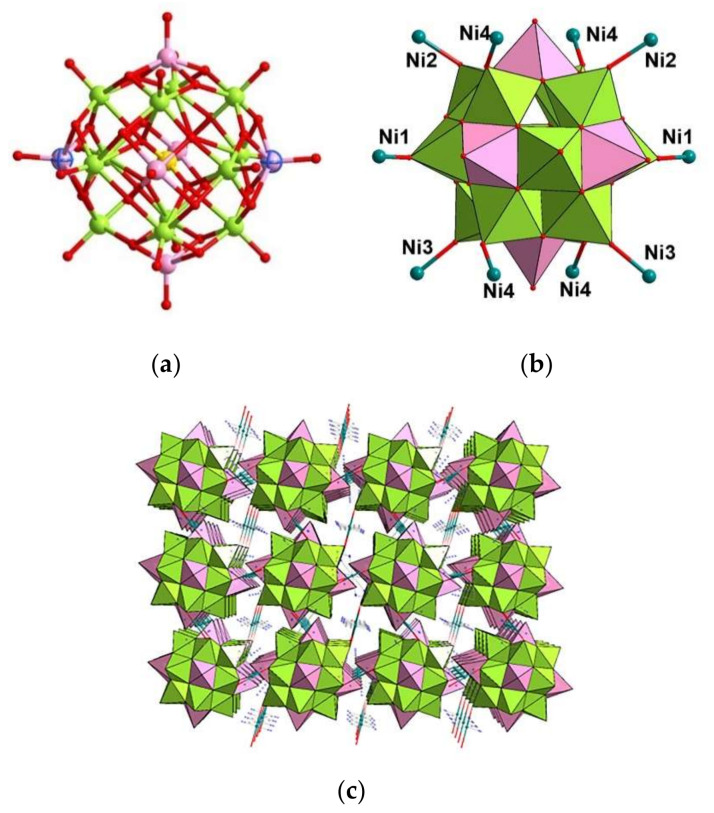
Crystal structure of **1**. (**a**) Ball-and-stick representation of {PNb_12_O_40_(VO)_5_} in **1**. (**b**) Connections between {PNb_12_O_40_(VO)_5_} and nickel ions. (**c**) The 3D framework of **1** viewed along the *b* direction. Color codes: Nb, green; V, pink; Ni, dark green; P, yellow; O, red; N, blue; C, gray.

Compound **1** was prepared by the hydrothermal reaction of K_7_HNb_6_O_19_·13H_2_O, VOPO_4_·2H_2_O, NaVO_3_, Ni(CH_3_COO)_2_·4H_2_O, and en at 160 °C. The single-crystal structural analysis (Table S1) reveals that **1** crystallizes in the monoclinic *C2/c* space group. Compound **1** is a 3D organic-inorganic framework constructed from [PNb_12_O_40_(VO)_5_]^5−^ ({PNb_12_V_5_}) clusters and ten [Ni(en)_2_]^2+^ linkers. The C_2h_ symmetric {PNb_12_V_5_} cluster contains a typical Keggin-type [PNb_12_O_40_]^15−^ ({PNb_12_}) capped by five {VO} units (Figure 1a). In the {PNb_12_} cluster, the centered heteroatom P connects with four edge-sharing {Nb_3_O_13_} subunits by sharing μ_4_-O atoms; the P–O_c_ (O_c_ = central oxygen) bond lengths are in the range of 1.539–1.550 Å and the O–P–O angles are in the range of 108.7–110.6°. Each Nb center is six-coordinated with octahedral geometry and the bond lengths are 1.730–1.768 Å for Nb–O_t_ (O_t_ = terminal oxygen), 1.912–2.035 Å for Nb–O_b_ (O_b_ = bridge oxygen), and 2.539–2.583 Å for Nb–O_c_. There are six square windows on {PNb_12_}, which are capped by four 100% occupied {VO} and two 50% occupied {VO} (front ellipses style) (Figure 1a). The penta-capped Keggin-type {PNb_12_O_40_(VO)_5_} in compound **1** presents a new V-containing PONb skeleton. All of the V centers are coordinated with four μ_2_-O atoms from {PNb_12_} and one terminal oxygen atom. The bond lengths of V–O_b_ are 1.929–2.340 Å, and those of V–O_t_ are 1.605–1.748 Å. According to the bond-valence sum (BVS) calculation, the five-capped V atoms are all in +4 oxidation state (Table S6). The oxidation state of the V atoms was further confirmed by XPS measurement. In the XPS spectrum of **1** (Figure S5), the peaks at 523.2 eV and 516.0 eV are attributable to V^4+^ 2p_1/2_ and V^4+^ 2p_3/2_, respectively.

There are four crystallographically independent Ni centers in **1** (Figure 1b); each one is coordinated with four N atoms from two en ligand and two terminal O atoms from two adjacent {PNb_12_O_40_(VO)_5_}. The Ni–N distances are in the range of 2.084–2.118 Å and the Ni–O_t_ distances are from 2.080 Å to 2.154 Å. Each {PNb_12_O_40_(VO)_5_} cluster was connected by ten adjacent [Ni(en)_2_]^2+^ linkers to form a 3D framework (Figure 1c). To our knowledge, it represents the first extended V-containing PONb connected by Ni-complex units. 

**Figure 2:**
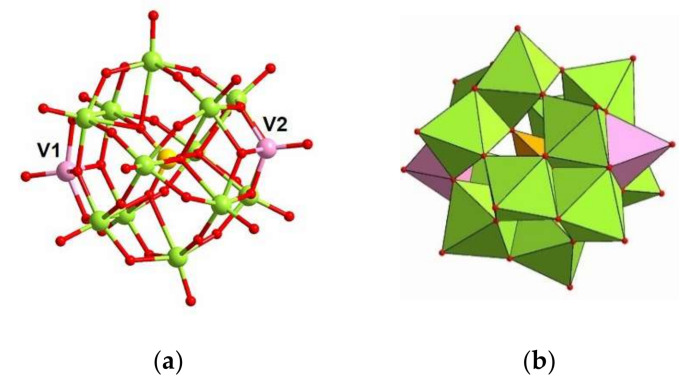
Crystal structure of polyanion in **2**. (**a**) Ball-and-stick representation of {PNb_12_O_40_(VO)_2_} in **2**. (**b**) Polyhedral representation of {PNb_12_O_40_(VO)_2_} in **2**. Color codes: Nb, green; V, pink; P, yellow; O, red.

When a similar hydrothermal reaction was performed at 140 °C without adding NaVO_3_, compound **2** was obtained. Compound **2** crystallizes in the orthorhombic *Pna2_1_* space group. Compound **2** contains a C_2v_ symmetric discrete [PNb_12_O_40_(VO)_2_]^10−^ ({PNb_12_V_2_}) cluster and five [Ni(en)_3_]^2+^ units as counter cations (Scheme 1). The polyanion {PNb_12_V_2_} features a {PNb_12_} cluster capping two {VO} units (Figure 2). As with **1**, the centered heteroatom P is coordinated with four μ_4_-O atoms, and all of the Nb centers are six-coordinated with octahedral geometry. The P–O_c_ bond lengths are in the range of 1.549–1.555 Å, and the O–P–O angles are in the range of 108.4–111.3°. The bond lengths of Nb–O_t_, Nb–O_b_, and Nb–O_c_ in {PNb_12_V_2_} are in the ranges 1.743–1.776 Å, 1.888–2.125 Å, and 2.498–2.604 Å, respectively. Two {VO} units are located on two opposite square windows on the surface of {PNb_12_}, and each V center exhibits square pyramidal geometry. The bond lengths of V–O_t_ and V–O_b_ are in the ranges 1.620–1.623 Å and 1.853–1.979 Å, respectively. Five free [Ni(en)_3_]^2+^ are distributed around {PNb_12_V_2_} and the Ni–N bond lengths are in the range of 2.061–2.164 Å. The XPS spectrum (Figure S6) indicates that there are both V^+4^ and V^+5^ oxidation states in **2**. In the V 2p region of **2**, the peaks at 523.4 eV and 515.8 eV are assigned to +4 oxidation state, and the peaks at 524.8 eV and 517.3 eV are attributable to +5 oxidation state, which are consistent with the BVS values of V (3.98 for V1 and 4.64 for V2) (Table S7).

We systematically explored the factors that influence the synthesis of **1** and **2**. It is found that the used en can effectively protect Ni^2+^ from hydrolysis under alkaline conditions. When 1,2-diaminopropane or 1,3-diaminopropane was used instead of en, the amount of precipitation was obtained. In addition, control experiments show that temperature and vanadium source play important roles in the synthesis of **1** and **2**. Following a procedure similar to that of **1**, compound **2** was obtained by removing NaVO_3_ at 140 °C. In addition, compound **2** cannot be obtained by lowering the hydrothermal temperature or varying the ratio of VOPO_4_ to NaVO_3_ in the synthesis of **1**. Therefore, we speculate that the evaluated hydrothermal temperature and the use of NaVO_3_ might increase the number of vanadium caps in the PONb cluster, and meanwhile the terminal O atoms of the Keggin-type {PNb_12_} would be activated by introducing additional vanadyl caps. As a result, two-capped {PNb_12_O_40_(VO)_2_} was isolated as a discrete cluster with the Ni-complex as counter cations, and five-capped {PNb_12_O_40_(VO)_5_} gave rise to a 3D framework by using the Ni-complex as linker. 

The IR spectra of **1** and **2** (Figure S2) were recorded from 4000 to 400 cm^−1^. The terminal M = O_t_ (M = Nb, V) vibrations are at 942 and 870 cm^−1^ for **1**, and at 947 and 877 cm^−1^ for **2**. The peaks at 805, 700, 627, 498, and 473 cm^−1^ of **1**, and at 815, 709, 638, 505, and 469 cm^−1^ of **2** are attributed to the bridging M–O–M vibrations. The peaks at 1032 cm^−1^ for **1**, and at 1027 cm^−1^ for **2**, are attributed to P–O_c_ vibration. The peaks at 1642–1048 cm^−1^ for **1,** and at 1593–1035 cm^−1^ for **2** can be assigned to the en ligand. In addition, for the two compounds, the broad band above 3000 cm^−1^ is attributed to the O–H vibrations of water molecules and/or the hydroxyl groups. 

**Figure 3:**
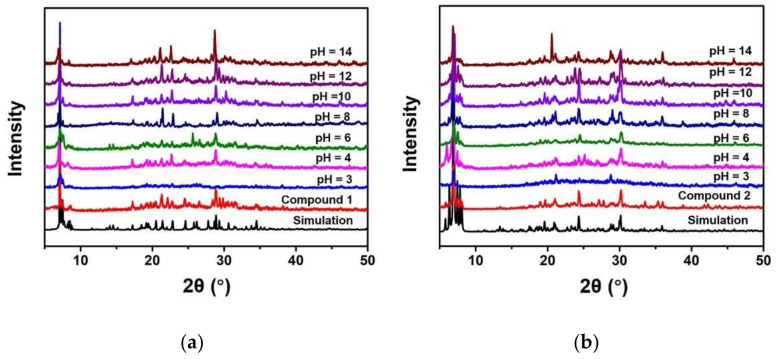
PXRD patterns of **1** (**a**) and **2** (**b**) after being soaked in aqueous solutions with different pH values for 24 h.

The phase purity of **1** and **2** was confirmed by PXRD (Figure S3), where the collected diffraction peaks match well with the simulated ones. Compounds **1** and **2** are nearly insoluble in water and common organic solvents, such as CH_2_Cl_2_, THF, CH_3_COCH_3_, CH_3_CN, and DMF (Figures S8 and S9). Therefore, we tested their pH stability in aqueous solution modified by PXRD (Figure 3) and IR spectra (Figure S7). As shown in Figure 3, **1** and **2** remained stable in the pH range of 4–14 after soaking for 24 h and began to decompose when the solution pH was 3. In addition, the crystals of **1** and **2** can keep their structure integrity after heating in organic solvent in the temperature range of 40–80 °C (Figures S10 and S11).

### 3.2. Electrocatalytic Selective Oxidation of Benzyl Alcohol

The selective oxidation of alcohols to aldehydes is one of the important organic transformations [43,44]. Compared with the traditional oxidation processes, the electrochemical oxidation provides an efficient and sustainable alternative [45,46]. Driven by electricity, alcohols can be oxidized on the anodic electrode under ambient conditions with hydrogen released from the cathodic electrode. Although some electrocatalysts have been developed in the anodic oxidation of alcohols, the selective oxidation of alcohols to the corresponding aldehydes remains a challenge, and in the reported system, the reaction activity significantly relies on the addition of alkaline additives [36,47,48]. Therefore, it is necessary to develop efficient and cost-effective electrode materials to realize the selective oxidation of alcohols under alkaline additive free conditions. 

**Figure 4:**
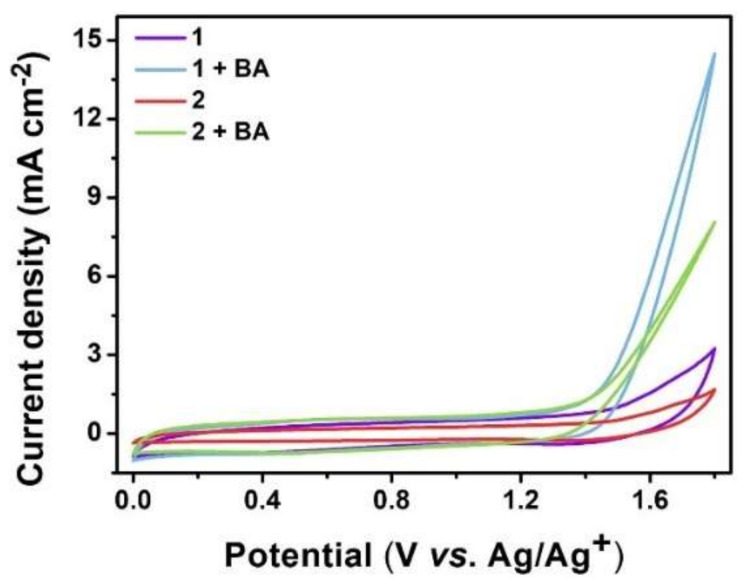
CV curves of **1** and **2** with BA (0.5 mmol) and without BA, obtained with a carbon cloth working electrode modified with **1** or **2** at a scan rate of 40 mV s^−1^.

**Figure 5:**
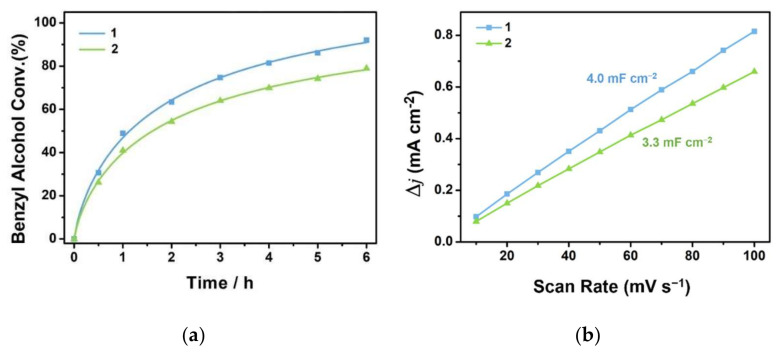
(**a**) Time profile for the electrocatalytic oxidation of BA by **1** and **2**; (**b**) The scan-rate dependence of current density at potential = 0.07 V vs. Ag/Ag^+^ for **1** and **2**.

Considering that V-containing PONbs **1** and **2** both have both Brønsted basicity and redox activity, we investigate the electrocatalytic activities of **1** and **2** using the selective oxidation of benzyl alcohol (BA) to benzaldehyde as a model reaction. The electrocatalytic activity of **1** and **2** was first evaluated by the cyclic voltammetry (CV) method, which was performed in an acetonitrile solution containing LiClO_4_ and BA with a carbon cloth modified by **1** or **2** as the working electrode. As shown in Figure 4, for **1** and **2**, the addition of BA leads to the significant increase in the anodic peak currents, indicating that the two compounds have a fast electrocatalytic response to the oxidation of BA. Notably, the anodic peak current of **1** is obviously higher than that of **2**, revealing that **1** has better electrocatalytic performance than **2**. The electrocatalytic activities of **1** and **2** were further verified by bulk electrolysis experiments performed in an undivided cell using **1** or **2** modified carbon cloth as the working electrode. As shown in Figure 5a and Table 1, both **1** and **2** are active for the selective oxidation of BA. Under ambient conditions, 92% of BA was converted by **1** in 6 h at the potential of 1.6 V vs. Ag/Ag^+^ and the selectivity for benzaldehyde reached 95%, giving the Faradaic efficiency (FE) of 93% (Table 1, entry 2). In addition, a trace amount of N-benzylacetamide as the only by-product was detected (Figures S12 and S14). Under the otherwise identical conditions, the catalytic activity of **2** (conversion: 79%, selectivity: 90%, FE: 84%, Table 1, entry 3) is lower than that of **1**. During the electrolysis process, hydrogen was released on the counter electrode (Figure S13).

To investigate the influence of the Ni-complex unit, PONb, and the capped V of **1** and **2** on the electrocatalytic selective oxidation of BA, the following control experiments were carried out (Table 1, entries 4–8). As shown in Table 1, entry 4, the electrocatalytic activity of the Ni-complex unit is negligible, because the catalytic performance of Ni(en)_3_Cl_2_ (conversion: 48%, selectivity: 69%, FE: 70%) is almost comparable to that of the blank test (conversion: 42%, selectivity: 73%, FE: 66%, Table 1, entry 1). When carbon cloth modified by K_7_H[Nb_6_O_19_]·13H_2_O was used as a working electrode, the conversion of BA (72%) was improved relative to the blank test, but the selectivity (79%) for benzaldehyde was still common (Table 1, entry 5). Moreover, a similar result was obtained by [N(CH_3_)_4_]_10_H_5_[PNb_12_O_40_] (conversion: 70%, selectivity: 78%, FE: 76%, Table 1, entry 6). The above results indicate that PONbs contribute to the conversion of BA because basic PONbs might facilitate the dehydrogenation oxidation of BA. Therefore, the temperature-programmed desorption of the carbon dioxide (CO_2_-TPD) measurement for **1** and **2** was performed, where the desorption peaks at 152 °C for **1** and 148 °C for **2** corresponding to the weak base site were observed, respectively (Figure S15). In addition, polyoxovanadate, K_6_[V_10_O_28_], can convert 83% of the substrate (Table 1, entry 7), but its selectivity (76%) is lower than that of the V-containing PONb **1** or **2**. As shown in Table 1, entry 8, the catalytic activity of the bicapped Keggin-type [N(CH_3_)_4_]_9_[PNb_12_O_40_(VO)_2_] (conversion: 74%, selectivity: 89%) is similar to that of **2** (conversion: 79%, selectivity: 90%). The control experiments above show that both the PONb cluster and the V caps contribute to the enhancement of BA oxidation. Then, we speculate that the different catalytic activity of **1** and **2** is mainly caused by their different number of V caps. This is further confirmed by the electrochemical surface area (ECSA) measurement: the ECSA of **1** (4.0 mF∙cm^−2^) with five V caps is higher than that of **2** (3.3 mF∙cm^−2^) with two V caps (Figure 5b and Figure S16).

**Figure 6:**
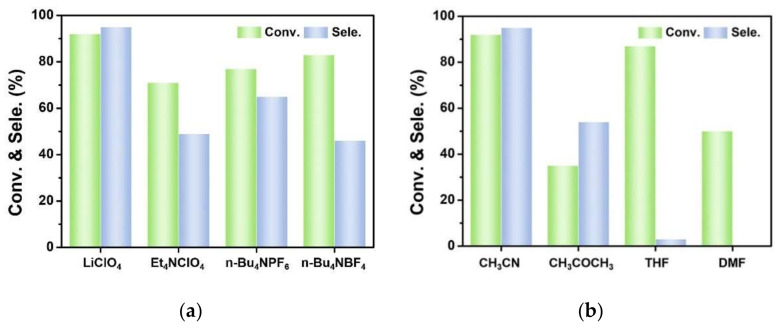

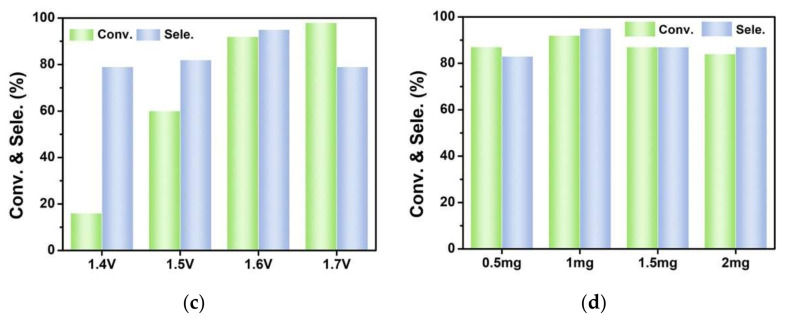
The influence of supporting electrolytes (**a**), solvent (**b**), applied potential (**c**), and catalyst dosage (**d**) on the electrocatalytic oxidation of BA by **1**.

To explore the optimal reaction conditions, we systematically investigated the influences of electrolyte, solvent, applied potential, and catalyst dosage on the electrocatalytic selective oxidation of BA by **1**. As shown in Figure 6a, compared with other types of supporting electrolytes, **1** exhibits excellent catalytic performance by using LiClO_4_. Meanwhile, it is found that acetonitrile with excellent conductivity exhibits a better performance than that of acetone, tetrahydrofuran, and N,N-dimethylformamide (Figure 6b). When the applied potential was increased from 1.4 to 1.6 V vs. Ag/Ag^+^, the conversion of BA was increased from 16% to 92%, but when it reached 1.7 V vs. Ag/Ag^+^, the selectivity decreased to 79%, although 98% of the BA was converted (Figure 6c). Therefore, 1.6 V vs. Ag/Ag^+^ is the optimal potential. As shown in Figure 6d, the best catalytic performance was achieved by using 1.0 mg **1**. After that, the catalytic activity was not further improved by increasing the catalyst dosage.

**Figure 7:**
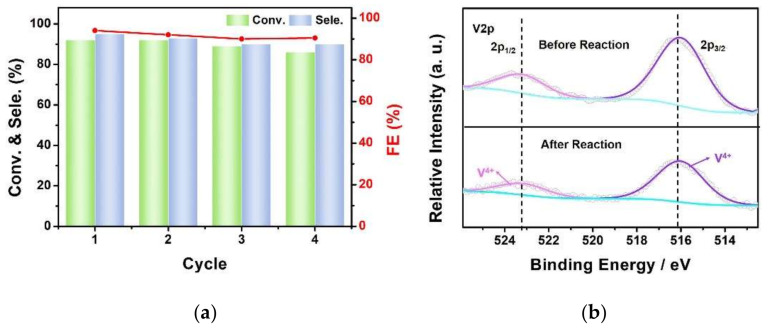
(**a**) Recycle test for the electrocatalytic selective oxidation of BA by **1**; (**b**) V 2p XPS spectra of **1** before and after the oxidation.

Moreover, the recyclability and stability of **1** were evaluated. As shown in Figure 7a, the catalytic activity of **1** is basically maintained after four cycles. There is no obvious change observed in the IR spectra of 1 before and after the reaction (Figure S17), revealing that compound **1** is basically stable after the recycle test. We compared the XPS spectra of **1** before and after the recycle. As shown in Figure 7b, in the V 2p region, the peaks at 523.2 eV and 516.0 eV are basically unchanged, indicating that the oxidation state of V remains +4 in **1**. Meanwhile, to verify the heterogeneity of **1**, the reaction solution was tested by ICP-OES (detection limit ca. 1 ppm) and no Nb, V, or Ni was detected, indicating that there is no catalyst leaching during the electrocatalytic process. In addition, no characteristic absorption of V-containing PONb is detected by the UV-vis spectrum (Figure S18).

In order to explore the possible mechanism of the electrocatalytic selective oxidation of BA by 1, we performed free radical trapping experiments. Oxygen radical scavenger, diphenylamine, and hydroxyl radical scavenger, *tert*-butanol, were added to the reaction system, respectively. As shown in Table S8, the oxidation of BA was significantly inhibited after the addition of diphenylamine. Therefore, we speculate that a free radical process was involved in the electrocatalytic oxidation of BA. Based on the experimental results, a plausible reaction mechanism was proposed (Figure S19). First, the electrocatalyst 1 in the reduced state (1-Red) is oxidized to its oxidized state (1-Ox) at the anode under constant potential. Then, the hydroxyl group of BA might be activated by the surface bridging O of the Keggin-type PONb cluster due to its Brønsted basicity [49,50]. After that, the BA is oxidized by 1-Ox through a −1e^−^/−1H^+^ process, generating the oxygen radical species (PhCH_2_O^•^). PhCH_2_O^•^ is further oxidized to benzaldehyde through another −1e^−^/−1H^+^ process, and meanwhile, 1-Ox is reduced to 1-Red, releasing protons to complete a catalytic cycle. The released protons are reduced at the cathode to produce H_2_.

## 4. Conclusions

In summary, two novel vanadium-containing Keggin-type PONbs modified by a Ni-complex have been successfully synthesized by controlling temperature and the vanadium source. In **1**, the five-capped {PNb_12_O_40_(VO)_5_} were connected by [Ni(en)_2_]^2+^ into a 3D framework; in **2**, the discrete bicapped {PNb_12_O_40_(VO)_2_} was isolated with [Ni(en)_3_]^2+^ as counter cations. Compounds **1** and **2** as organic-inorganic hybrid materials exhibit good pH stability in aqueous solution and thermal stability in organic solvent. Importantly, under alkaline additive free conditions, compounds **1** and **2** are highly active for the selective oxidation of BA. The control experiments show that both the Brønsted basicity of PONb and the redox activity of the V caps play an important role in the electrocatalytic process. This study not only enriches the structure data base of vanadium-containing PONbs but also extends their catalytic application.

## Figures and Tables

**Table 1:** Electrocatalytic selective oxidation of benzyl alcohol by different catalysts ^a^.

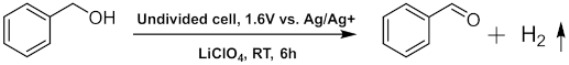				
Entry	Cat.	Conv. (%) ^b^	Sele. (%) ^b^	FE (%)
1	-	42	73	66
2	Compound **1**	92	95	93
3	Compound **2**	79	90	84
4	Ni(en)_3_Cl_2_·H_2_O	48	69	70
5	K_7_H[Nb_6_O_19_]·13H_2_O	72	79	76
6	[N(CH_3_)_4_]_10_H_5_[PNb_12_O_40_]·30.5H_2_O	70	78	76
7	K_6_[V_10_O_28_]·10H_2_O	83	76	78
8	[N(CH_3_)_4_]_9_[PNb_12_O_40_(VO)_2_]·19H_2_O	74	89	82

^a^ Standard reaction conditions: CH_3_CN (10 mL), LiClO_4_ (1.0 mmol), BA (0.5 mmol), reaction time: 6 h, constant potential: 1.6 V vs. Ag/Ag^+^. ^b^ The product conversion and selectivity were determined by GC analysis using biphenyl as internal standard.

## Data Availability

Data are contained within the article and supplementary materials.

## Supplementary Materials

CCDC 2164660 (**1**), 2164659 (**2**) contains the supplementary crystallographic data for this paper. These data can be obtained free of charge via http://www.ccdc.cam.ac.uk/conts/retrieving.html, accessed on 13 April 2022. The following supporting information can be downloaded at: https://www.mdpi.com/article/10.3390/molecules27092862/s1, Table S1: Crystal data and structure refinement for **1** and **2**; Tables S2–S5: Selected bond lengths and bond angles for **1** and **2**; Tables S6 and S7: BVS results of Ni and V atoms for **1** and **2**; Table S8: Electrocatalytic oxidation of benzyl alcohol (BA) catalyzed by **1** in the presence of radical scavengers; Figure S1: Digital photographs of **1** and **2**; Figure S2: the IR spectra of **1** and **2**; Figure S3: the PXRD patterns of **1** and **2**; Figure S4: The TG curves of **1** and **2**; Figure S5: The XPS spectra for Ni(2p) and V(2p) in **1**; Figure S6: The XPS spectra for Ni(2p) and V(2p) in **2**; Figure S7: The IR spectra of **1** and **2** after being soaked in aqueous solutions with different pH values for 24 h; Figures S8 and S9: The PXRD patterns and IR spectra of **1** and **2** after being soaked in different solvents; Figures S10 and S11: The PXRD patterns and IR spectra of **1** and **2** after heating in acetonitrile for 2 h; Figure S12: Gas chromatogram of the benzyl alcohol oxidation by **1**; Figure S13: The gas chromatograph of benzyl alcohol oxidation before and after reaction; Figure S14: GC-MS spectrum of the by-product; Figure S15: CO_2_-TPD for **1** and **2**; Figure S16: CV curves of **1** and **2** at different scan rates; Figure S17: The IR spectra of **1** after the recycle test; Figure S18: UV-vis spectra of postreaction solution, BA, benzyl aldehyde, and PNb_12_V_2_; Figure S19: Proposed mechanism for the electrocatalytic selective oxidation of BA to benzaldehyde by **1**.

## Sample Availability

Samples of compounds **1** and **2** are available from the authors.
