# Supplementary material for: Controllable Assembly of Vanadium-Containing Polyoxoniobate-Based Materials and Their Electrocatalytic Activity for Selective Benzyl Alcohol Oxidation
Source: Molecules. 2022 Apr 30;27(9):2862. doi: 10.3390/molecules27092862 (PMC9101498; doi:10.3390/molecules27092862)
Supplement: Supplementary file 1 [file molecules-27-02862-s001.zip › molecules-1705450-supplementary.pdf]

Supplementary Materials

Controllable Assembly of Vanadium-Containing Polyoxoniobate-Based Materials and their Electrocatalytic Activity for Selective Benzyl Alcohol Oxidation

Xiaoxia Li, Ni Zhen, Chengpeng Liu, Di Zhang, Jing Dong, Yingnan Chi*, Changwen Hu*

Key Laboratory of Cluster Science of Ministry of Education, Beijing Key Laboratory of Photoelectronic/Electrophotonic Conversion Materials, School of Chemistry and Chemical Engineering, Beijing Institute of Technology, Beijing 102488, China; 3120191243@bit.edu.cn(X.L.); zhenni1220@163.com (N.Z.); laucouple@163.com (C.L.); dizhang_pub@163.com (D.Z.); 20200808@btbu.edu.cn (J.D.)

* Correspondence: chiyingnan7887@bit.edu.cn (Y.C.); cwhu@bit.edu.cn (C.H.)

Single-Crystal X-ray Crystallography.

Crystallographic data for compounds **1** and **2** were performed on a Bruker APEX-II DUO CCD single-crystal diffractometer with graphite-monochromatized Mo Kα radiation (λ= 0.71073 Å) at 296 K. The crystals were solved by direct methods and refined by full-matrix least squares method on F^2 using the SHELXL-2018/6 program. The H atoms on lattice water, hydroxyl ion and organic ligand, are not located. All heavy atoms were refined with anisotropic thermal parameters. The crystal data and structure refinement results are summarized in Table S1. The selected bond lengths (Å) and angles (deg) of compounds **1**-**2** are listed in Table S2-S5. BVS results for compounds **1** and **2** are listed in Table S6 and S7. The crystallographic data have been deposited with the Cambridge Crystallographic Data Centre (CCDC) as entries 2164660 (**1**) and 2164659 (**2**).

According to the single crystal X-ray diffraction data, there are some residual disordered or partial-occupied water and hydroxyl ions are not located in the cif file. And the crystal water and hydroxyl ions cannot be modeled with accurate location due to the weak diffraction, which is very common in POM chemistry. Meanwhile, due to the diffraction difference between the heavy Nb atoms and light H atoms, the H atoms on lattice water, hydroxyl ions and organic ligand are very difficult to model. Final molecular formula was determined by single-crystal X-ray diffraction data, elemental analysis and TG analysis. Therefore, the calculated Mol. weight and Mu Ratio differs from reported ones, and the density is different, too. In addition, low bond precision on C-C bonds may due to the moderate data quality in context with Nb as heavy atoms.

Table S1. Crystal data and structure refinement for compounds **1** and **2**.

	**1**	**2**
empirical formula	$PNb_{12}V_5Ni_5O_{68}C_{20}H_{121}N_{20}$	$PNb_{12}V_2Ni_5O_{59}C_{30}H_{154}N_{30}$
FW (g·mol^{-1})	3424.50	3420.91
temperature (K)	296.15	296(2)
wavelength (Å)	0.71073	0.71073
cryst syst	monoclinic	orthorhombic
space group	*C2/c*	$Pna2_1$
a (Å)	20.5209(18)	30.509(2)
b (Å)	20.1530(17)	15.6335(11)
c (Å)	21.3606(19)	22.5687(16)
α (deg)	90	90
β (deg)	93.702(2)	90
γ (deg)	90	90
volume (Å^3)	8815.4(13)	10764.6(13)
Z	2	4
density calcd (g·cm^{-3})	1.290	2.111
abs coeff, mm^{-1}	1.586	2.355
F(000)	3374.0	6836.0
index ranges	$-24 \le h \le 24$, $-23 \le k \le 23$, $-25 \le l \le 25$	$-36 \le h \le 36$, $-18 \le k \le 18$, $-27 \le l \le 27$
reflns collected	40246	105079
indep reflns	7758	19730
GOF on F^2	1.068	1.203
final *R* indices	$R_1^a = 0.0745$	$R_1^a = 0.0362$
$[I >= 2\sigma(I)]$	$wR_2^b = 0.2361$	$wR_2^b = 0.1140$
R indices (all data)	$R_1^a = 0.0807$	$R_1^a = 0.0378$
	$wR_2^b = 0.2436$	$wR_2^b = 0.1165$

$^aR_1 = \Sigma||F_0| - |F_c|| / \Sigma|F_0|$;

$^bwR_2 = \Sigma[w(F_0^2\text{-}F_c^2)^2]/ \Sigma[w(F_0^2)^2]^{1/2}$

Table S2. Selected bond lengths of **1**.

Bond type	Bond length	Bond type	Bond length	Bond type	Bond length
P1-O17	1.550(7)	Nb4-O20	1.963(7)	V3-O7	1.938(7)
P1-O23	1.539(7)	Nb4-O23_a	2.583(7)	V4-O8	2.038(8)
Nb1-O10	2.006(7)	Nb5-O4	2.003(7)	V4-O9	1.929(7)
Nb1-O11	1.920(7)	Nb5-O9	1.979(7)	V4-O10	1.975(8)
Nb1-O13	1.768(8)	Nb5-O14	2.007(7)	V4-O18	2.129(9)
Nb1-O17	2.540(7)	Nb5-O16	1.730(7)	V4-O24	1.644(10)
Nb1-O18	2.003(7)	Nb5-O17	2.525(7)	Ni1-O13_2	2.127(8)
Nb1-O22	1.978(8)	Nb5-O18	1.948(7)	Ni1-O13	2.127(8)
Nb2-O6_a	2.033(7)	Nb6-O6	1.948(7)	Ni2-O19_c	2.128(7)
Nb2-O7	2.026(7)	Nb6-O7	1.994(7)	Ni2-O19	2.128(7)
Nb2-O11	1.950(7)	Nb6-O8	1.989(7)	Ni3-O21	2.098(7)
Nb2-O20	1.964(7)	Nb6-O10	1.955(7)	Ni3-O21_d	2.098(7)
Nb2-O21	1.736(7)	Nb6-O12	1.731(7)	Ni4-O12_e	2.154(7)
Nb2-O23_a	2.558(7)	Nb6-O23	2.560(7)	Ni4-O16	2.080(7)
Nb3-O4_a	2.035(7)	V1-O5	1.748(19)	Ni1-N10	2.096(10)
Nb3-O14	2.026(7)	V1-O11	1.975(9)	Ni1-N9	2.118(9)
Nb3-O15	1.912(7)	V1-O15	1.932(9)	Ni2-N7	2.088(11)
Nb3-O17	2.539(7)	V1-O20	2.217(10)	Ni2-N8	2.112(11)
Nb3-O19	1.758(7)	V1-O22	2.340(10)	Ni3-N5	2.116(11)
Nb3-O22	1.941(7)	V2-O2	1.605(12)	Ni3-N6	2.097(10)
Nb4-O1	1.737(8)	V2-O4	1.950(7)	Ni4-N1	2.084(9)
Nb4-O8_a	2.004(7)	V2-O14	2.044(7)	Ni4-N2	2.093(10)
Nb4-O9_a	2.012(7)	V3-O3	1.608(13)	Ni4-N3	2.084(9)
Nb4-O15	1.957(7)	V3-O6	2.040(7)	Ni4-N4	2.103(9)

Symmetry transformations used to generate equivalent atoms: a = 1-X,+Y,3/2-Z; b = 1/2-X,1/2-Y,1-Z; c = 1-X,1-Y,1-Z; d = 1-X,-Y,1-Z; e = 1/2-X,1/2+Y,3/2-Z.

Table S3. Selected bond angles of **1.**

Bond type	Bond angle	Bond type	Bond angle	Bond type	Bond angle
O17-P1-O17_a	109.2(6)	N8_c-Ni2-N8	180.0	N1-Ni4-N2	84.1(4)
O23-P1-O17	109.8(4)	N7_c-Ni2-O19	90.3(3)	N1-Ni4-N3	95.2(4)
O23-P1-O17_a	108.7(4)	N7-Ni2-O19	89.7(3)	N1-Ni4-N4	178.0(3)
O23-P1-O23_a	110.6(6)	N8_c-Ni2-O19	90.6(3)	N2-Ni4-N4	96.4(4)
N9_b-Ni1-N9	180.0	N8-Ni2-O19	89.4(3)	N3-Ni4-N2	178.5(3)
N10_b-Ni1-N9	97.1(4)	O19_c-Ni2-O19	180.0	N3-Ni4-N4	84.3(4)
N10-Ni1-N9	82.9(4)	N5_d-Ni3-N5	180.0	N1-Ni4-O12_e	90.2(3)
N10_b-Ni1-N10	180.0(5)	N5_d-Ni3-N6	96.3(4)	N2-Ni4-O12_e	90.7(3)
N9-Ni1-O13_b	91.8(3)	N6-Ni3-N5	83.7(4)	N3-Ni4-O12_e	90.6(3)
N9-Ni1-O13	88.2(3)	N6^{4}-Ni3-N6	180.0	N4-Ni4-O12_e	91.7(3)
N10-Ni1-O13	91.8(3)	N6-Ni3-O21	89.0(4)	N1-Ni4-O16	90.6(3)
N10-Ni1-O13_b	88.2(3)	N6-Ni3-O21_d	91.0(4)	N2-Ni4-O16	88.1(3)

O13_b-Ni1-O13	180.0	N5-Ni3-O21_d	88.5(4)	N3-Ni4-O16	90.6(3)
N7_c-Ni2-N7	180.0	N5-Ni3-O21	91.5(4)	N4-Ni4-O16	87.4(3)
N7-Ni2-N8	84.4(4)	N21_d-Ni3-O21	180.0	O16-Ni4-O12_e	178.4(3)
N7_c-Ni2-N8	95.6(4)				

Symmetry transformations used to generate equivalent atoms: a = 1-X,+Y,3/2-Z; b = 1/2-X,1/2-Y,1-Z; c = 1-X,1-Y,1-Z; d = 1-X,-Y,1-Z; e = 1/2-X,1/2+Y,3/2-Z.

Table S4. Selected bond lengths of **2**.

Bond type	Bond length	Bond type	Bond length	Bond type	Bond length
P1-O9	1.555(7)	Nb6-O42	2.091(6)	V1-O18	1.961(6)
P1-O10	1.551(7)	Nb7-O4	1.759(7)	V1-O31	1.620(8)
P1-O35	1.555(6)	Nb7-O5	2.086(7)	V1-O42	1.979(6)
P1-O36	1.549(7)	Nb7-O9	2.519(6)	V2-O5	1.863(6)
Nb1-O1	2.075(6)	Nb7-O22	1.895(7)	V2-O6	1.623(6)
Nb1-O10	2.506(7)	Nb7-O39	2.118(7)	V2-O7	1.954(6)
Nb1-O18	2.104(7)	Nb7-O40	1.906(7)	V2-O33	1.853(7)
Nb1-O19	1.923(7)	Nb8-O10	2.597(7)	V2-O39	1.979(6)
Nb1-O21	1.760(7)	Nb8-O19	2.012(7)	Ni1-N1	2.151(12)
Nb1-O32	1.899(7)	Nb8-O27	1.979(7)	Ni1-N2	2.144(12)
Nb2-O2	1.750(7)	Nb8-O28	1.995(7)	Ni1-N21	2.122(13)
Nb2-O3	1.967(7)	Nb8-O29	1.743(7)	Ni1-N22	2.139(12)
Nb2-O9	2.556(7)	Nb8-O37	1.973(7)	Ni1-N27	2.164(11)
Nb2-O30	2.010(7)	Nb9-O7	2.106(7)	Ni1-N28	2.103(12)
Nb2-O32	1.976(7)	Nb9-O14	1.903(7)	Ni2-N3	2.119(12)
Nb2-O40	1.994(7)	Nb9-O25	1.753(7)	Ni2-N4	2.124(10)
Nb3-O1	2.089(6)	Nb9-O27	1.896(7)	Ni2-N5	2.125(12)
Nb3-O3	1.912(7)	Nb9-O33	2.125(7)	Ni2-N23	2.114(12)
Nb3-O20	1.764(7)	Nb9-O36	2.511(6)	Ni2-N24	2.141(12)
Nb3-O26	1.904(7)	Nb10-O9	2.548(7)	Ni2-N26	2.153(11)
Nb3-O35	2.530(6)	Nb10-O30	1.895(7)	Ni3-N8	2.118(12)
Nb3-O42	2.090(7)	Nb10-O33	2.110(7)	Ni3-N9	2.106(13)
Nb4-O12	1.763(8)	Nb10-O34	1.776(7)	Ni3-N10	2.128(11)
Nb4-O13	1.973(7)	Nb10-O37	1.887(7)	Ni3-N11	2.137(14)
Nb4-O14	1.998(7)	Nb10-O39	2.114(7)	Ni3-N12	2.150(13)
Nb4-O16	1.959(7)	Nb11-O8	1.775(7)	Ni3-N25	2.145(13)
Nb4-O36	2.604(6)	Nb11-O10	2.510(6)	Ni4-N13	2.103(13)
Nb4-O38	1.996(7)	Nb11-O16	1.902(7)	Ni4-N14	2.139(13)
Nb5-O5	2.109(7)	Nb11-O17	2.094(6)	Ni4-N15	2.138(14)
Nb5-O7	2.119(6)	Nb11-O18	2.091(7)	Ni4-N16	2.101(13)
Nb5-O23	1.775(7)	Nb11-O28	1.901(7)	Ni4-N17	2.100(11)
Nb5-O24	1.902(7)	Nb12-O15	1.754(7)	Ni4-N18	2.099(14)
Nb5-O36	2.516(7)	Nb12-O22	1.982(7)	Ni5-N6	2.114(15)
Nb5-O38	1.885(7)	Nb12-O24	1.975(7)	Ni5-N7	2.072(15)

Nb6-O11	1.763(7)	Nb12-O26	1.997(7)	Ni5-N19	2.092(16)
Nb6-O13	1.888(7)	Nb12-O35	2.596(7)	Ni5-N20	2.101(17)
Nb6-O17	2.094(6)	Nb12-O41	1.988(7)	Ni5-N29	2.066(17)
Nb6-O35	2.498(6)	V1-O1	1.891(7)	Ni5-N30	2.061(19)
Nb6-O41	1.900(7)	V1-O17	1.876(7)		

Table S5. Selected bond angles of **2**.

Bond type	Bond angle	Bond type	Bond angle	Bond type	Bond angle
O9-P1-O10	109.1(4)	N4-Ni2-N26	170.3(5)	N16-Ni4-N13	82.7(5)
O9-P1-O35	108.4(4)	N5-Ni2-N24	93.0(5)	N16-Ni4-N14	93.3(5)
O9-P1-O36	110.3(3)	N5-Ni2-N26	90.7(4)	N16-Ni4-N15	93.5(5)
O10-P1-O35	111.1(3)	N23-Ni2-N3	91.4(5)	N17-Ni4-N13	93.1(5)
O10-P1-O36	109.3(4)	N23-Ni2-N4	94.8(4)	N17-Ni4-N14	91.6(5)
O35-P1-O36	108.7(4)	N23-Ni2-N5	173.2(5)	N17-Ni4-N15	92.8(5)
N1-Ni1-N27	95.9(5)	N23-Ni2-N24	82.0(5)	N17-Ni4-N16	172.4(6)
N2-Ni1-N1	81.0(5)	N23-Ni2-N26	94.0(4)	N18-Ni4-N13	96.5(6)
N2-Ni1-N27	94.1(5)	N24-Ni2-N5	91.5(4)	N18-Ni4-N14	90.6(5)
N21-Ni1-N1	172.2(5)	N8-Ni3-N10	91.6(5)	N18-Ni4-N15	172.1(6)
N21-Ni1-N2	94.1(5)	N8-Ni3-N11	93.0(5)	N18-Ni4-N16	91.7(5)
N21-Ni1-N22	82.1(5)	N8-Ni3-N12	94.9(5)	N18-Ni4-N17	82.4(5)
N21-Ni1-N27	90.5(5)	N8-Ni3-N25	81.6(5)	N7-Ni5-N6	81.6(6)
N22-Ni1-N1	92.2(5)	N9-Ni3-N8	172.6(5)	N7-Ni5-N19	96.1(6)
N22-Ni1-N2	95.1(5)	N9-Ni3-N10	82.9(5)	N7-Ni5-N20	92.0(6)
N22-Ni1-N27	168.5(5)	N9-Ni3-N11	92.4(5)	N19-Ni5-N6	177.4(6)
N28-Ni1-N1	92.8(5)	N9-Ni3-N12	91.0(5)	N19-Ni5-N20	85.4(6)
N28-Ni1-N2	171.1(5)	N9-Ni3-N25	93.8(5)	N20-Ni5-N6	95.9(6)
N28-Ni1-N21	92.7(5)	N10-Ni3-N11	94.2(5)	N29-Ni5-N6	91.6(6)
N28-Ni1-N22	91.5(5)	N10-Ni3-N12	172.1(5)	N29-Ni5-N7	172.9(7)
N28-Ni1-N27	80.0(4)	N10-Ni3-N25	95.2(5)	N29-Ni5-N19	90.7(7)
N3-Ni2-N4	93.7(5)	N11-Ni3-N12	80.9(5)	N29-Ni5-N20	90.7(7)
N3-Ni2-N5	94.1(5)	N11-Ni3-N25	169.3(5)	N30-Ni5-N6	91.2(7)
N3-Ni2-N24	170.5(5)	N25-Ni3-N12	90.3(5)	N30-Ni5-N7	89.0(7)
N3-Ni2-N26	82.0(5)	N13-Ni4-N14	171.9(6)	N30-Ni5-N19	87.6(7)
N4-Ni2-N5	80.8(4)	N13-Ni4-N15	90.0(5)	N30-Ni5-N20	173.0(7)
N4-Ni2-N24	93.6(4)	N15-Ni4-N14	83.2(5)	N30-Ni5-N29	89.2(7)

Table S6. BVS results of Ni and V atoms of compound **1**.

Atom	Value	Atom	Value	Atom	Value
Ni1	1.89	Ni2	1.91	Ni3	1.94
Ni4	1.97	V1	3.21	V2	3.89
V3	3.93	V4	3.63		

Table S7. BVS results of Ni and V atoms of compound **2**.

Atom	Value	Atom	Value	Atom	Value
Ni1	1.84	Ni2	1.88	Ni3	1.88
Ni4	1.96	Ni5	2.13	V1	3.98
V2	4.64				

Table S8. Electrocatalytic oxidation of benzyl alcohol (BA) catalyzed by **1** in the presence of radical scavengers [a].

Entry	Scavenger (0.5mmol)	Conversion (%)	Selectivity (%)
1	diphenylamine	30	28
2	*tert*-butanol	92	84

[a] Reaction conditions: BA (0.5 mmol), CH_3CN (10 mL), $LiClO_4$ (1.0 mmol), reaction time: 6 h, constant potential: 1.6 V vs. Ag/Ag^+.

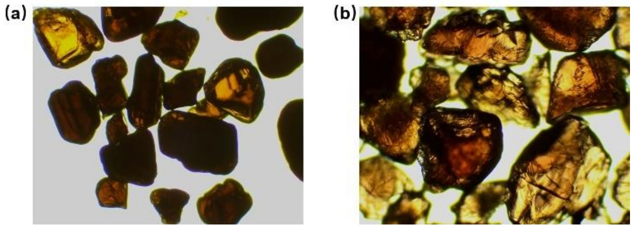

Figure S1. Digital photographs of **1** (a) and **2** (b).

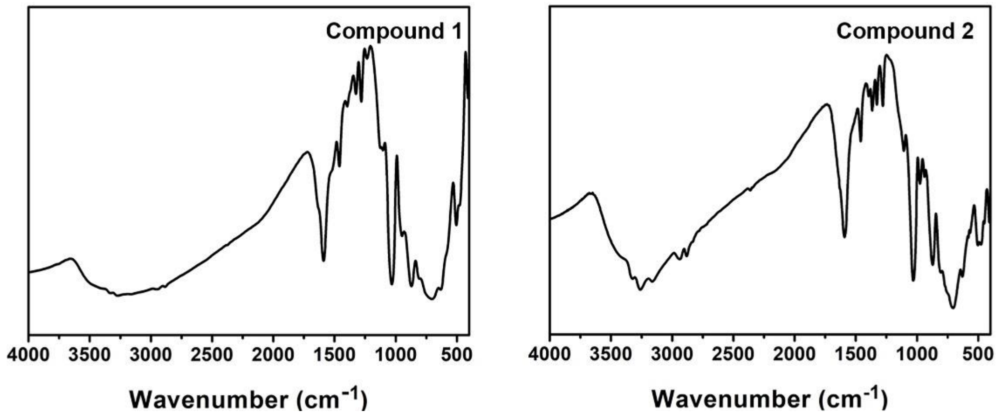

Figure S2. The IR spectra of **1** and **2**.

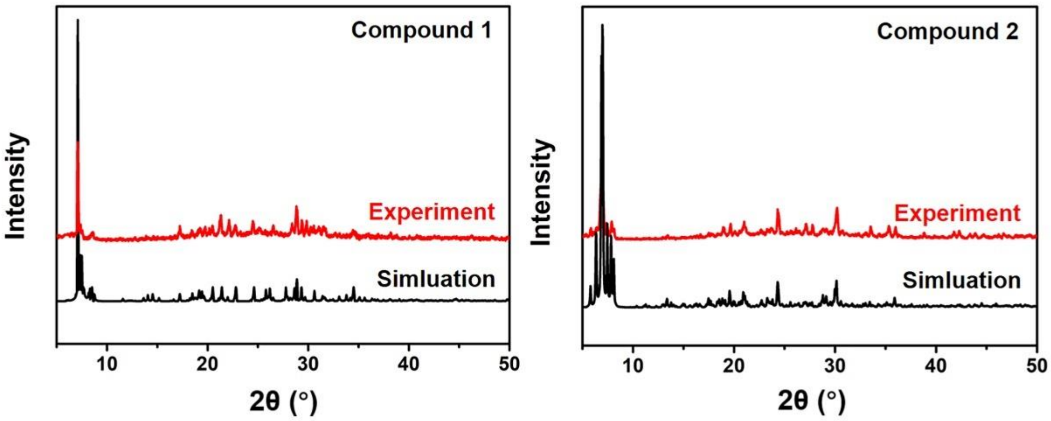

Figure S3. The PXRD patterns of **1** and **2**.

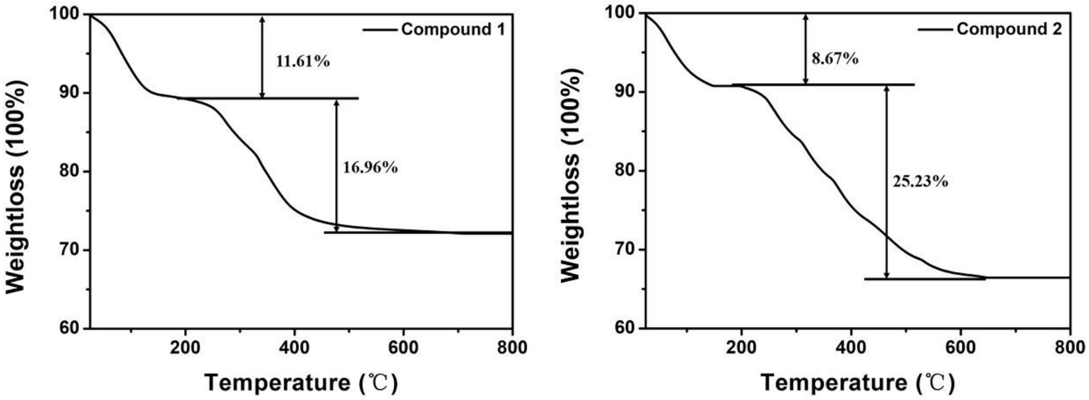

Figure S4. The TG curves of **1** and **2** measured from 30 to 800 °C under N_2 atmosphere. The TG curves of **1** and **2** show two continuous weight loss stages, which are fallen in the regions about 25-700 °C. In the TG curve of **1**, the first step of weight loss is 11.61% (calc. 11.94%) in the range of 25-200 °C can be assigned to the loss of eighteen lattice water molecules and five hydroxyl ions. The second weight loss of 16.96% (calc. 17.55%) from 200 to 700 °C is correspond to the decomposition of ten en molecules. In the TG curve of **2**, the first weight loss from 25 to 200 °C is attributed to the loss of seventeen lattice water molecules (found 8.67%, calc. 8.94%), and the other weight loss of 25.23% (calc. 26.01%) from 200 to 700 °C is correspond to the decomposition of en molecules.

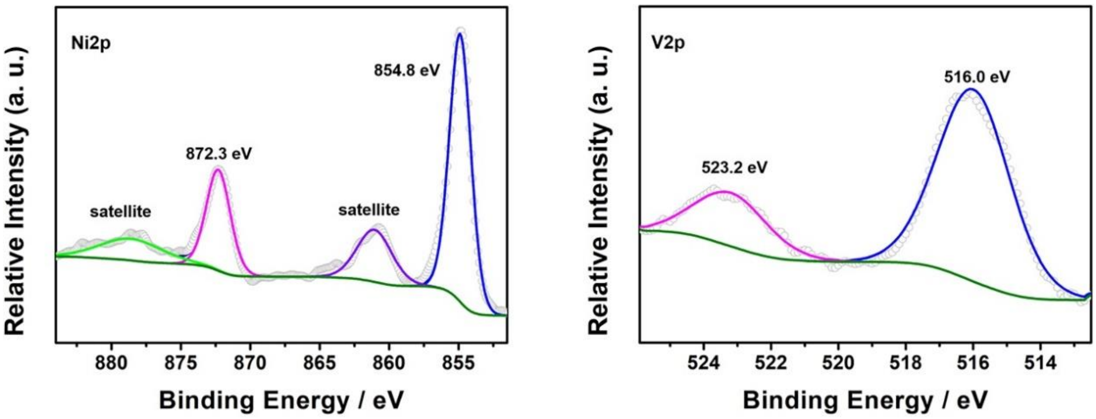

Figure S5. The XPS spectra for Ni(2p) and V(2p) in **1**.

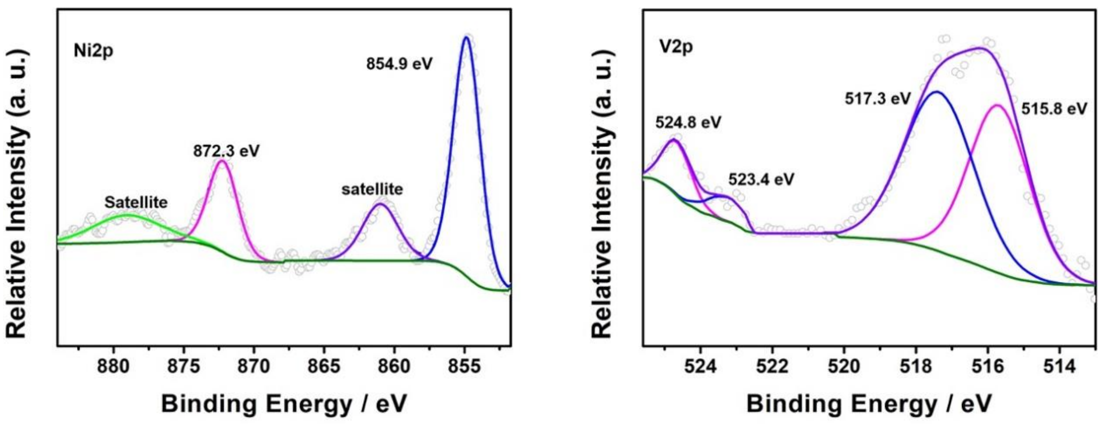

Figure S6. The XPS spectra for Ni(2p) and V(2p) in **2**.

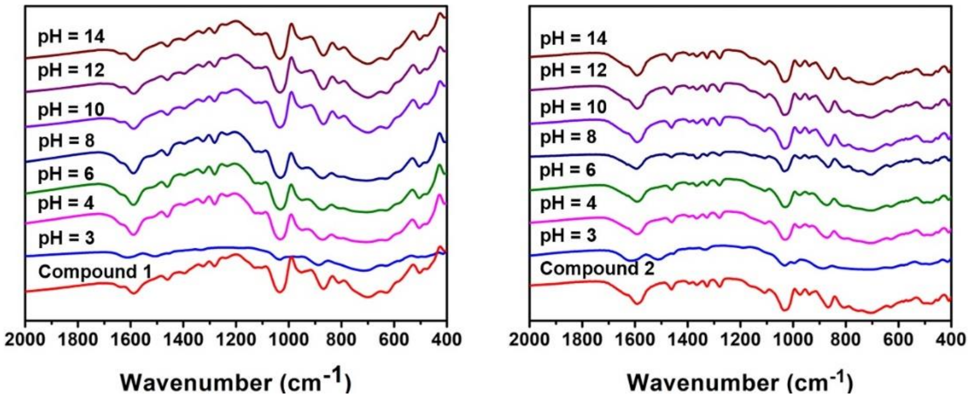

Figure S7. The IR spectra of **1** (left) and **2** (right) after being soaked in aqueous solutions with different pH values for 24 h.

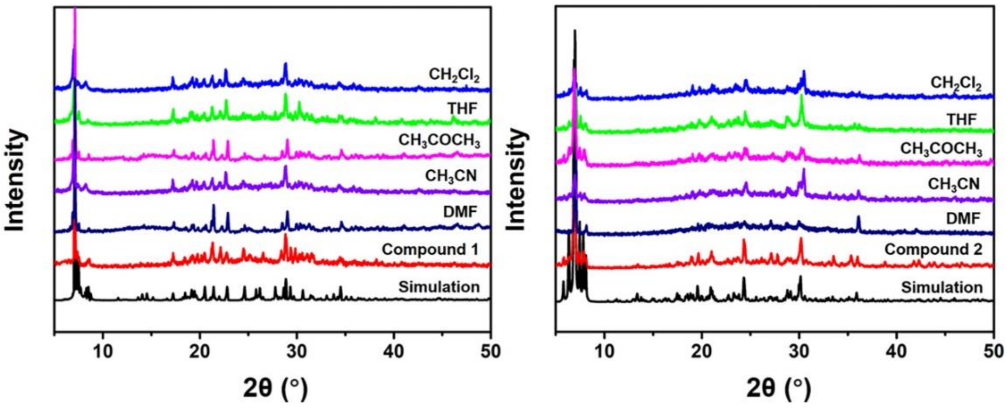

Figure S8. The PXRD patterns of **1** (left) and **2** (right) after being soaked in different organic solvents.

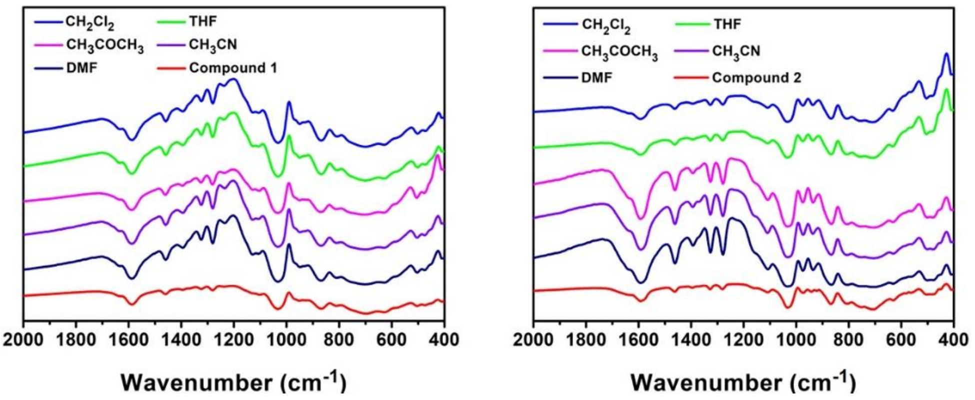

Figure S9. The IR spectra of **1** (left) and **2** (right) after being soaked in different organic solvents.

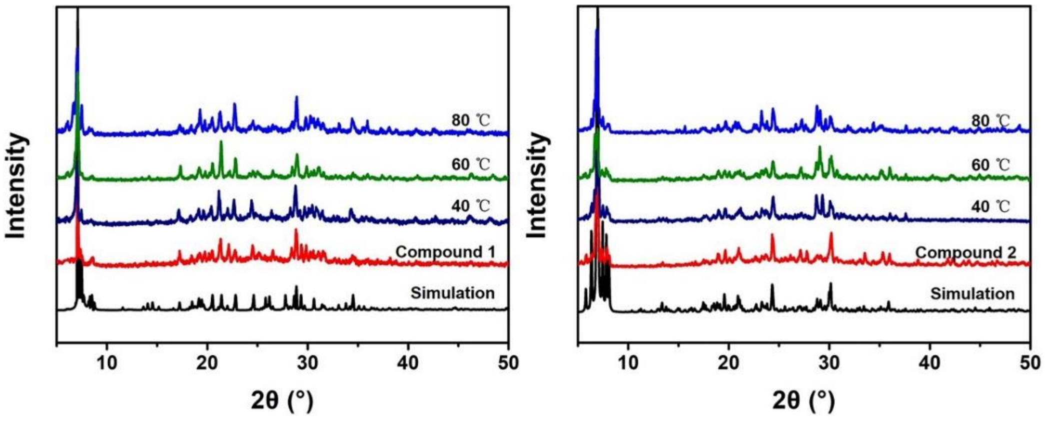

Figure S10. The PXRD patterns of **1** (left) and **2** (right) after heating in acetonitrile for 2 h.

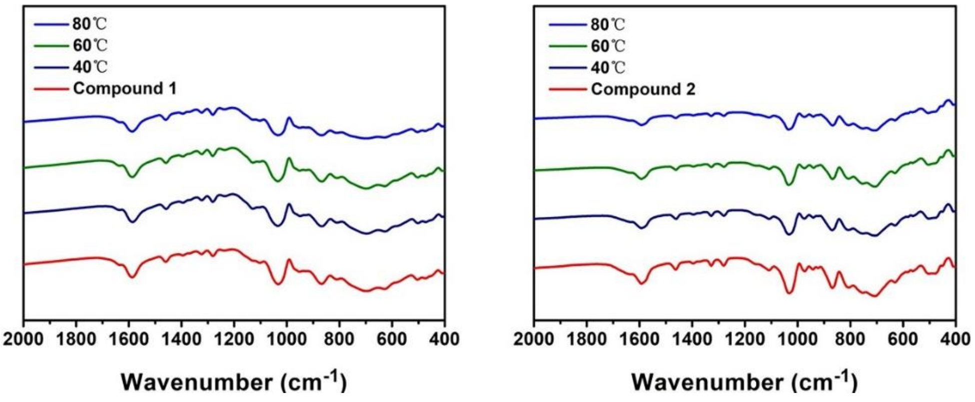

Figure S11. The IR spectra of **1** (left) and **2** (right) after heating in acetonitrile for 2 h.

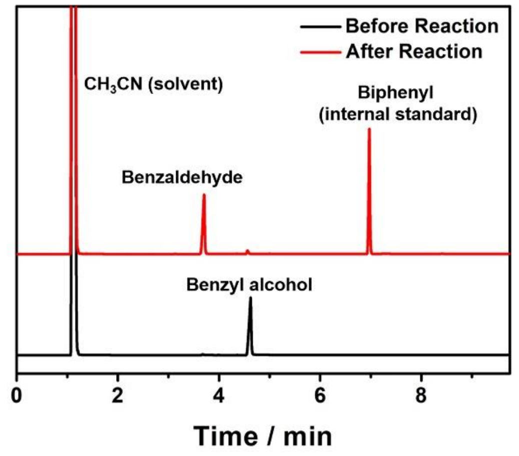

Figure S12. Gas chromatogram of the benzyl alcohol oxidation by **1**.

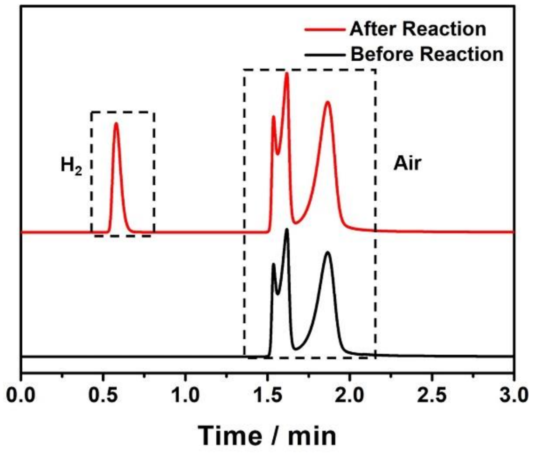

Figure S13. The gas chromatograph of benzyl alcohol oxidation before and after reaction. The peak at 0.57 min is assigned to H_2.

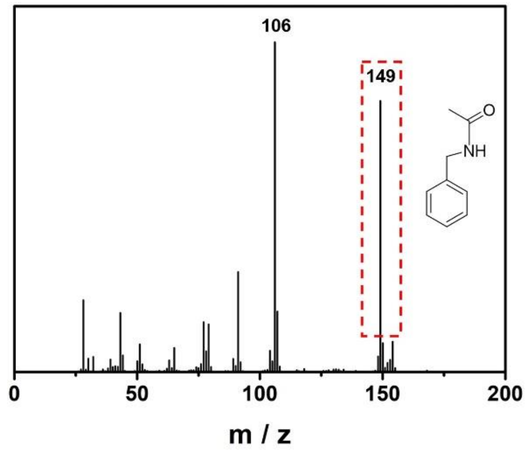

Figure S14. GC-MS spectrum of the by-product N-benzylacetamide.

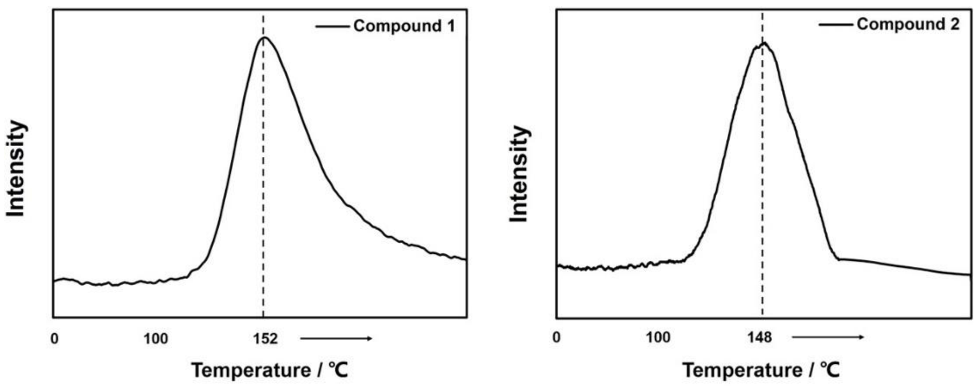

Figure S15. CO_2-TPD for **1** and **2**.

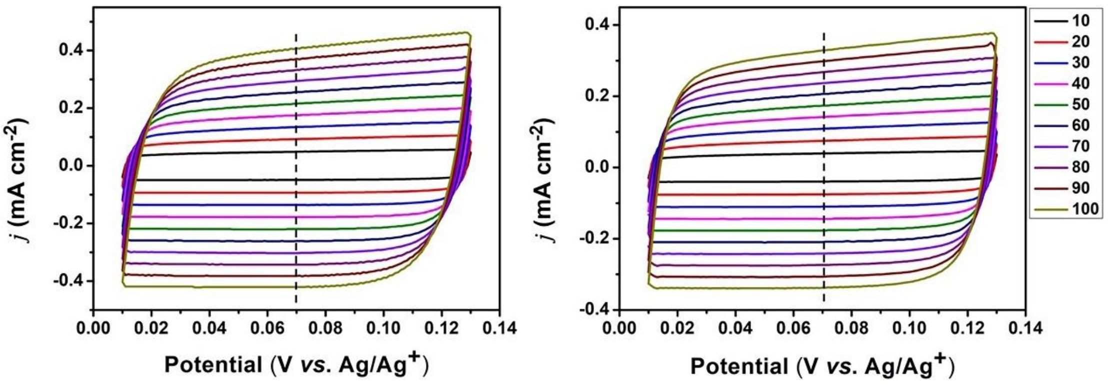

Figure S16. CV curves of **1** and **2** at different scan rates of in a potential window where no Faradaic processes occur (0.01-0.13 V vs. Ag/Ag^{+}).

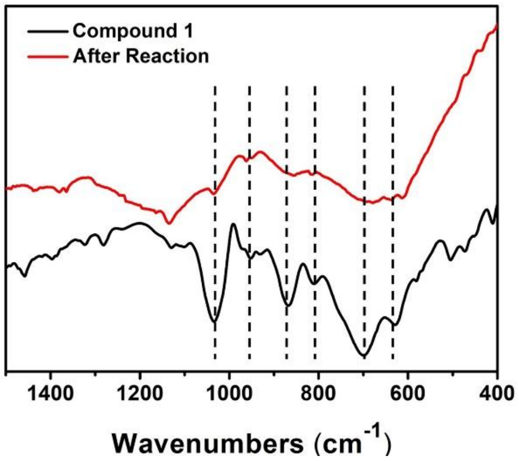

Figure S17. The IR spectra of **1** before and after the recycle test.

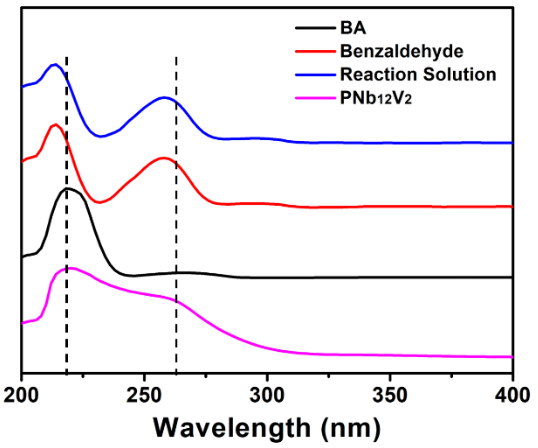

Figure S18. UV-vis spectra of postreaction solution, BA, benzyl aldehyde, and $PNb_{12}V_2$.

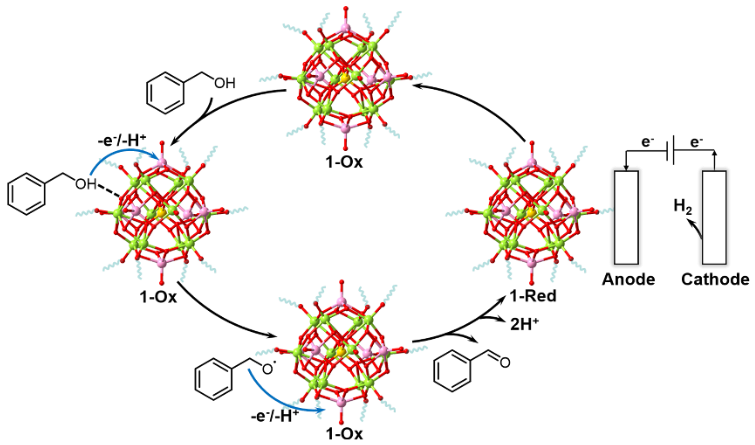

Figure S19. Proposed mechanism for the electrocatalytic selective oxidation of BA to benzaldehyde by **1**.
